# Seminal Plasma Exosomal miRNA Profiling Reveals hsa‐miR‐7‐5p as a Key Regulator of Sperm Motility in Asthenozoospermia

**DOI:** 10.1111/andr.70159

**Published:** 2025-12-24

**Authors:** Zhi‐Jian Zhu, Meng‐Li Cui, Wen‐Jing Yuan, Jia‐Ning Zhang, Shuo Zhang, Xi‐Qiao Yao, Ruo‐Qing Li, Jin‐Feng Li, En‐Zhong Li

**Affiliations:** ^1^ Affiliated Central Hospital Huanghuai University Zhumadian China; ^2^ College of Biological and Food Engineering Huanghuai University Zhumadian China; ^3^ Topfond Pharmaceutical Co., Ltd Zhumadian China

**Keywords:** asthenospermia, male infertility, miRNAs, RAF1, seminal plasma exosomes

## Abstract

**Background:**

Asthenozoospermia is a significant contributing factor to male infertility. Accumulating evidence indicates that impaired sperm motility is closely linked to dysregulated microRNA expression during spermatogenesis. Seminal plasma exosomes are enriched with diverse microRNAs, which play pivotal roles in modulating sperm motility, the acrosome reaction, capacitation, and fertilization.

**Objective:**

To investigate the association between exosomal microRNA profiles and sperm motility by comparing microRNA expression patterns in seminal plasma exosomes from asthenozoospermic patients and healthy controls, thereby elucidating the molecular mechanisms underlying exosomal microRNA‐mediated regulation of sperm motility.

**Materials and Methods:**

Seminal plasma exosomes were isolated from 21 asthenozoospermic patients and 21 age‐matched healthy donors via ultracentrifugation, followed by characterization via transmission electron microscopy, nanoparticle tracking analysis, and Western blotting. Exosomal microRNA profiles were analyzed via unique molecular identifier‐based small RNA sequencing to identify motility‐associated microRNAs.

**Results:**

Comparative analysis revealed 13 significantly upregulated microRNAs in the exosomes of asthenozoospermic patients, among which hsa‐miR‐7‐5p exhibited the greatest abundance. Functional enrichment analysis revealed that the target genes of hsa‐miR‐7‐5p were associated primarily with cellular metabolism, macromolecule metabolic processes, and nitrogen compound metabolism, as well as several crucial signaling pathways, including mTOR signaling, endocrine resistance, and gonadotropin‐releasing hormone secretion. A dual‐luciferase reporter assay verified that hsa‐miR‐7‐5p directly targets rapidly accelerated fibrosarcoma 1, a pivotal component of the gonadotropin‐releasing hormone signaling pathway.

**Discussion and Conclusion:**

hsa‐miR‐7‐5p may regulate sperm motility through the rapidly accelerated fibrosarcoma 1 ‐mediated gonadotropin‐releasing hormone secretion and MAPK signaling networks. Our findings highlight the potential role of exosomal microRNAs in male infertility. Future prospective studies are warranted to elucidate the molecular mechanisms underlying the regulation of sperm motility.

## 1 Introduction

Spermatogenesis is a tightly regulated process in which spermatogonial stem cells undergo self‐renewal and differentiation to ultimately give rise to mature spermatozoa [1, 2, 3]. This intricate process relies on precise regulatory mechanisms, and any disruption can result in conditions such as azoospermia, asthenospermia (AS), and oligospermia, all of which are major contributors to male infertility [4, 5]. In recent years, a significant decline in semen quality has been observed among men, particularly in key parameters including sperm concentration, total sperm count, sperm motility, and normal morphology. Sperm motility is widely acknowledged as one of the critical determinants of male fertility, with AS accounting for approximately 30% of male infertility cases [6]. While conventional sperm motility analysis remains the diagnostic gold standard, it offers limited insights into the underlying molecular mechanisms. This gap hinders the development of effective diagnostic tools and therapeutic strategies for this prevalent condition.

Seminal plasma is rich in exosomes—membrane‐bound vesicles that constitute approximately 3% of the total protein content in seminal plasma [7]. It is crucial to recognize that seminal exosomes represent a heterogeneous population derived primarily from two distinct sources: prostasomes secreted by the prostate gland and epididymosomes secreted by the epididymis [8]. These exosomal subpopulations exhibit spatial dynamics throughout the male reproductive tract, with their composition and function varying according to their tissue origin and the specific microenvironment encountered by spermatozoa during maturation. Seminal plasma exosomes harbor a broad range of bioactive regulatory molecules, including lipids, microRNAs (miRNAs), mRNAs, and proteins, all of which play critical roles in sperm motility, the acrosome reaction, capacitation, and fertilization [9, 10, 11, 12]. Comparative proteomic analysis between normozoospermic and asthenozoospermic men revealed that five specific proteins are markedly absent in the exosomes of asthenozoospermic individuals [13]. Furthermore, seminal plasma exosomes isolated from normospermic men present significantly higher expression levels of CRISP3 than those from men with severe AS [14]. CRISP3, a protein crucial for sperm motility, is transferred from seminal plasma exosomes to sperm cells, thereby increasing their motility [15]. Glycodelin, an exosomal protein, was detected in samples from asthenospermic patients but was absent in normospermic individuals [16]. Additionally, recent studies have increasingly highlighted the expression of miRNAs in seminal exosomes and their regulatory effects on sperm motility [17, 18, 19].

Seminal plasma exosomes contain a significant number of miRNA molecules that play essential roles in regulating spermatogenesis, sperm maturation, and fertilization potential [17, 20]. Dysregulations in miRNA expression profiles have been linked to spermatogenic arrest, fertilization defects, and various reproductive disorders [21, 22]. Following testicular departure, sperm cells enter a transcriptionally quiescent state, halting autonomous miRNA biosynthesis. Instead, they acquire regulatory miRNAs through fusion with exogenous extracellular vesicles (EVs) primarily derived from the epididymis and prostate. This miRNA transfer mechanism reprograms the sperm miRNA landscape, which is critical for completing post‐testicular maturation and acquiring fertilization‐related functions [23, 24]. The functional interpretation of seminal exosomal miRNAs must account for their spatial origins. Exosomes secreted by cells from different regions of the epididymis contain miRNAs that exhibit substantial variations in both quantity and type [3], and these epididymal exosomal miRNAs are extensively involved in regulating sperm maturation and motility [25, 26]. Similarly, prostatic fluid constitutes a key component of semen, and prostasomes account for a considerable proportion of seminal plasma exosomes. Studies have shown that miR‐34c‐5p, miR‐122, miR‐513a‐5p, and miR‐509‐5p are highly expressed in prostasomes and contribute to promoting sperm maturation while inhibiting premature capacitation [27]. However, the admixture of exosomes from divergent origins within seminal plasma obscures their regional‐specific functions, thereby complicating the elucidation of their precise biological roles.

In this study, we utilized unique molecular identifier (UMI)‐based absolute quantification sequencing technology to analyze the differential expression profiles of miRNAs in seminal plasma exosomes derived from normal individuals and patients with asthenozoospermia, aiming to identify key miRNAs that potentially play functional roles beyond merely serving as biomarkers. Our findings revealed that the expression level of hsa‐miR‐7‐5p was significantly elevated in the seminal exosomes of asthenozoospermic patients compared with those of healthy controls. Furthermore, subsequent analysis indicated that hsa‐miR‐7‐5p might regulate sperm motility by interacting with the rapidly accelerated fibrosarcoma 1 (RAF1) protein, suggesting a potential mechanistic pathway through which exosomal miRNAs may influence sperm function.

## 2 Materials and Methods

### 2.1 Patient Enrollment

Twenty‐one asthenozoospermic men aged 18–48 years were recruited, fulfilling the following criteria: sperm concentration >15 × 10⁶/mL, progressive motility (grade a + b) <32%, and total motility <40%. Furthermore, 21 age‐matched healthy controls with normal semen parameters and confirmed fertility were included. The sperm parameters of asthenozoospermic patients and normal controls are shown in File S1. Participants with varicocoele, urogenital infections, reproductive tract infections, or a history of smoking were excluded. All procedures adhered to the principles of the Declaration of Helsinki and were approved by the institutional ethics committee, and written informed consent was obtained from all participants.

### 2.2 Isolation of Exosomes From Human Seminal Plasma

Fresh semen samples were collected from each participant after ≥3 days of sexual abstinence and allowed to liquefy at 37°C for 30 min. Within 2 h of collection, the samples were centrifuged at 1500×*g* for 10 min to separate spermatozoa from seminal plasma. EVs are a heterogeneous population, with exosomes defined as small EVs (sEVs) (40–150 nm) and microvesicles (MVs) as larger EVs (100–800 nm). Due to their overlapping size ranges, ultracentrifugation‐based isolation typically enriches for exosomes while potentially coisolating smaller MVs. To minimize contamination from larger EVs, the seminal plasma was processed through sequential centrifugation: first at 10,000×*g* at 4°C for 30 min to remove cellular debris and large particles, followed by centrifugation at 20,000×*g* for 90 min to pellet MVs. Finally, ultracentrifugation at 200,000×*g* for 120 min at 4°C was performed to obtain exosome‐rich pellets. The exosomal pellets were washed with phosphate‐buffered saline (PBS), recentrifuged under identical conditions, resuspended in PBS, and stored at −80°C for subsequent analysis.

### 2.3 Transmission Electron Microscopy

Transmission electron microscopy (TEM) was employed to characterize the spherical morphology of the contents within the suspended pellet. Specifically, the diluted sample was adsorbed onto a carbon‐coated copper grid for 60 s. The excess solution was subsequently blotted away carefully, and the sample was negatively stained with 2% uranyl acetate for 1 min. Finally, the samples were analyzed via a Hitachi H‐7700 transmission electron microscope.

### 2.4 Nanoparticle Tracking Analysis

The samples were processed in duplicate and diluted with PBS to achieve a concentration of 10–100 particles per image before analysis. The average diameter and concentration were automatically measured via a ZetaView Nanoparticle Tracker.

### 2.5 Exosomal RNA Isolation and RNA Analysis

Total RNA for UMI absolute quantification transcriptome sequencing was extracted via TRIzol Reagent (Life Technologies, Carlsbad, CA, USA) according to the manufacturer's protocol. The RNA concentration was determined by measuring the absorbance at 260 nm (A260), and the purity was assessed by the A260/A280 ratio via a NanoDrop spectrophotometer (NanoDrop; Thermo Scientific, MA, USA). RNA integrity was evaluated by determining the RNA integrity number via a Bioanalyzer 2100 (Agilent Technologies, Santa Clara, CA, USA).

### 2.6 UMI‐Based Small RNA Library Construction and Sequencing

Small RNA libraries were prepared via the NEBNext Multiplex Small RNA Library Prep Kit (NEB; Cat# E7300S). Briefly, RNA was ligated to 3′ and 5′ adapters containing 8‐nt UMIs. UMI sequences were randomized to minimize PCR bias. First‐strand cDNA synthesis was performed via ProtoScript II Reverse Transcriptase (NEB) with UMI‐containing adapters. PCR amplification was optimized to avoid overamplification, followed by size selection (140–160 bp) on a 6% polyacrylamide gel. Finally, RNA‐seq data were obtained via the Illumina NovaSeq 6000 platform (PE150; 20 million reads/sample) by Novogene Co., Ltd. (Beijing, China). The clean reads obtained from the Silva, GtRNAdb, Rfam, and Repbase databases were aligned with Bowtie software. Next, ribosomal RNA, transfer RNA, small nuclear RNA, small nucleolar RNA, and other non‐coding RNAs and repeats were filtered out. The remaining reads were used to detect known miRNAs and novel miRNAs predicted via comparison with known miRNAs from miRBase. We further utilized the MirAligner module in the SeqBuster software to analyze canonical and other isomeric miRNA variants. Two technical replicate sequencing runs were performed for each pooled sample to ensure the technical robustness of the procedure.

### 2.7 Sample Pooling and Sequencing Strategy

To ensure sufficient exosomal RNA yield for library construction and to reduce inter‐individual variation for robust biomarker discovery, exosome samples within the same group were pooled. Specifically, equal volumes (100 µL) of exosomal samples from each of the 21 asthenozoospermic individuals were combined to form a single case pool. Similarly, equal volumes (100 µL) of exosomal samples from each of the 21 normozoospermic controls were combined to form a single control pool. This pooling strategy enhances the representation of consistent, group‐specific miRNA signatures while mitigating the impact of random individual fluctuations. Total RNA was extracted from the pooled exosome samples and subjected to RNA quality analysis as detailed in Section 2.5. Subsequently, two independent small RNA sequencing runs were subsequently performed, one for each pooled sample, utilizing the UMI‐based absolute quantification sequencing technology detailed in Section 2.6.

### 2.8 Cell Culture

HEK‐293T cells and the GC‐1 spermatogonia cell line were maintained in DMEM/F12 medium supplemented with 10% fetal bovine serum (FBS), 100 U/mL penicillin, and 100 mg/mL streptomycin. Primary murine epididymal epithelial cells (EECs) were isolated from 4‐week‐old mice. Briefly, the epididymides were aseptically harvested, minced, and sequentially digested with 0.25% trypsin for 5 min at 37°C, followed by 0.25% collagenase I for 10 min. The resulting cell suspension was filtered through a 100 µm mesh and resuspended in MEM medium containing 10% FBS, 1 mmol/L sodium pyruvate, 1 nmol/L dihydrotestosterone, 100 U/mL penicillin, and 100 mg/mL streptomycin. All cell types were cultured at 37°C in a humidified atmosphere with 5% CO_2_.

### 2.9 Reverse Transcription and Quantitative Real‐Time PCR

According to the sequencing results, four upregulated miRNAs, hsa‐miR‐3130‐3p, hsa‐miR‐378e, hsa‐miR‐7‐5p, and hsa‐miR‐454‐5p, were chosen as candidate miRNAs for further validation via relative quantitative real‐time qRT‐PCR. The small‐scale rescreening cohort, comprising 21 asthenospermic patients and 21 normal controls, underwent seminal exosome extraction using commercial kits (ExoQuick Exosome Extraction Kit; SBI) to validate the sequencing findings. Specific stem‒loop reverse transcription primers were used for cDNA synthesis of hsa‐miR‐3130‐3p, hsa‐miR‐378e, hsa‐miR‐7‐5p, hsa‐miR‐454‐5p, and MIR2911 with the PrimeScript II first Strand cDNA Synthesis Kit (TaKaRa). Real‐time PCR was conducted with the SYBR Premix Ex Taq II Kit (TaKaRa) on an Applied Biosystems StepOne Real‐time PCR system (Applied Biosystems). The Bulge‐Loop and qRT‐PCR primers were synthesized and procured from Shanghai GenePharma Co., Ltd. The PCRs were incubated at 95°C for 30 s, followed by 40 cycles of 95°C for 5 s and 60°C for 30 s. Melting curve analysis was performed via default methods. MIR2911 served as the exogenous reference gene for normalization. Three independent experiments were carried out, with each PCR performed in triplicate.

For the detection of RAF1 mRNA expression in sperm cells, total RNA was extracted from spermatozoa isolated from the same cohort of normozoospermic controls (*n* = 21) and asthenozoospermic patients (*n* = 21) using TRIzol Reagent. Reverse transcription was performed using the PrimeScript RT reagent Kit (TaKaRa), followed by qRT‐PCR with SYBR Premix Ex Taq II (TaKaRa) and specific primers for RAF1. The relative mRNA expression level was normalized to β‐actin (ACTB).

### 2.10 Target Gene Prediction and Enrichment Analysis

The bioinformatics software TargetScan (https://www.targetscan.org/vert_71/) was used to predict miRNA target genes. Gene Ontology (GO) term and Kyoto Encyclopedia of Genes and Genomes (KEGG) pathway enrichment analyses were performed to explore the biological functions of the target genes.

### 2.11 Dual‐Luciferase Reporter Assay

The miRNA target verification assays were performed in HEK293T cells. The wild‐type or mutant 3′UTR dual‐luciferase reporter (200 ng each) along with the hsa‐miR‐7‐5p or negative control (NC) (50 nM each) were cotransfected into HEK293T cells via the Lipofectamine 2000 reagent (Invitrogen) in 48‐well plates. At 48 h post‐transfection (hpt), the cells were washed twice with PBS (pH 7.4) and lysed with 20 µL of 1× passive lysis buffer for 15 min at room temperature. The activities of firefly and Renilla luciferase were measured via the Dual‐Luciferase Reporter Assay system (Promega, USA). All the data were acquired from three independent repeats and calculated as the means (M) ± standard deviations (SD) via the software “GraphPad Prism” (version 6.0).

### 2.12 Establishment of hsa‐miR‐7‐5p Overexpression Model in EECs

To investigate the functional role of hsa‐miR‐7‐5p in EECs, we established an in vitro overexpression model. Primary EECs isolated from 4‐week‐old mice as described in Section 2.8 were seeded in six‐well plates and cultured until reaching 60–70% confluence. The cells were then transfected with either hsa‐miR‐7‐5p mimics (50 nM) or NC mimics (50 nM) using Lipofectamine 2000 reagent (Invitrogen) according to the manufacturer's instructions. Transfection efficiency was confirmed by qRT‐PCR at 24 and 48 hpt. Cells were harvested at the indicated time points for subsequent RNA and protein extraction.

### 2.13 Western Blot Analysis

Sperm cells in liquefied semen were collected by density gradient centrifugation using 40 and 80% Percoll gradient reagents. The spermatozoa were washed with PBS, resuspended, and the concentration was adjusted to 1 × 10⁶–1 × 10⁷ cells/mL. The sperm cells and EECs were lysed for whole‐cell extracts with RIPA buffer (Solarbio, Beijing, China) supplemented with protease inhibitors (Roche, USA). The protein concentrations of the fractions were determined via a Micro BCA protein assay reagent kit (Thermo Fisher Scientific). The cell lysates with equal volumes of SDS‒PAGE loading buffer were run on 10% SDS‒PAGE gels and transferred to PVDF membranes. The membranes were blocked in 5% non‐fat milk and incubated with the corresponding protein‐specific primary antibodies overnight at 4°C, followed by incubation with HRP‐conjugated secondary antibodies for 2 h at room temperature. Luminescent signals were subsequently detected with Western blotting chemiluminescence reagent (Perkin Elmer, USA) and recorded with a Vilber Lourmat system (Vilber, Paris, France). The antibodies used are as follows: anti‐heat shock protein (Hsp)70 mouse monoclonal antibody (1:1000 dilution; Cat. ab2787, Abcam); anti‐Tsg101 rabbit monoclonal antibody (1:1000 dilution; Cat. ab133586; Abcam); anti‐Calnexin rabbit monoclonal antibody (1:1000 dilution; Cat. ab92573; Abcam); anti‐RAF1 rabbit monoclonal antibody (1:1000 dilution; Cat. ab181115; Abcam); anti‐ACTB mouse monoclonal antibody (1:1000 dilution; Cat. D190826; Sangon Biotech). At least three independent Western blots were performed for each experiment.

To analyze the endogenous protein expression of RAF1 in sperm cells, spermatozoa from the liquefied semen of normozoospermic controls (*n*  = 21) and asthenozoospermic patients (*n* = 21) were isolated by density gradient centrifugation as described above. The isolated sperm cells were subsequently lysed, and Western blotting was performed using the anti‐RAF1 antibody as outlined in this section.

### 2.14 Protein‒Protein Interaction Network Analysis

The screened target genes were imported into the search tool for the retrieval of interacting genes/proteins (STRING v10.0, http://string‐db.org/) via the selection of *Homo sapiens* as a model organism. A combined score >0.4 was used as the cut‐off criterion. The protein–protein interaction (PPI) networks were subsequently visualized via Cytoscape software (http://www.cytoscape.org), and the nodes with higher degrees of connectivity were taken as hub nodes.

### 2.15 Statistical Analysis

Unpaired two‐tailed Student's *t*‐tests were conducted via SPSS software to determine the statistical significance of the data obtained from different experimental groups. All graphs represent the M ± SD of normalized data points for triplicate samples from each of three independent repeats. “*” and “**” indicate statistically significant differences, with values of *p* < 0.05 and *p* < 0.01, respectively. The *p* value was corrected via the method of Benjamini and Hochberg to control the FDR.

## 3 Results

### 3.1 Isolation and Characterization of Exosomes From Human Seminal Plasma

**FIGURE 1:**
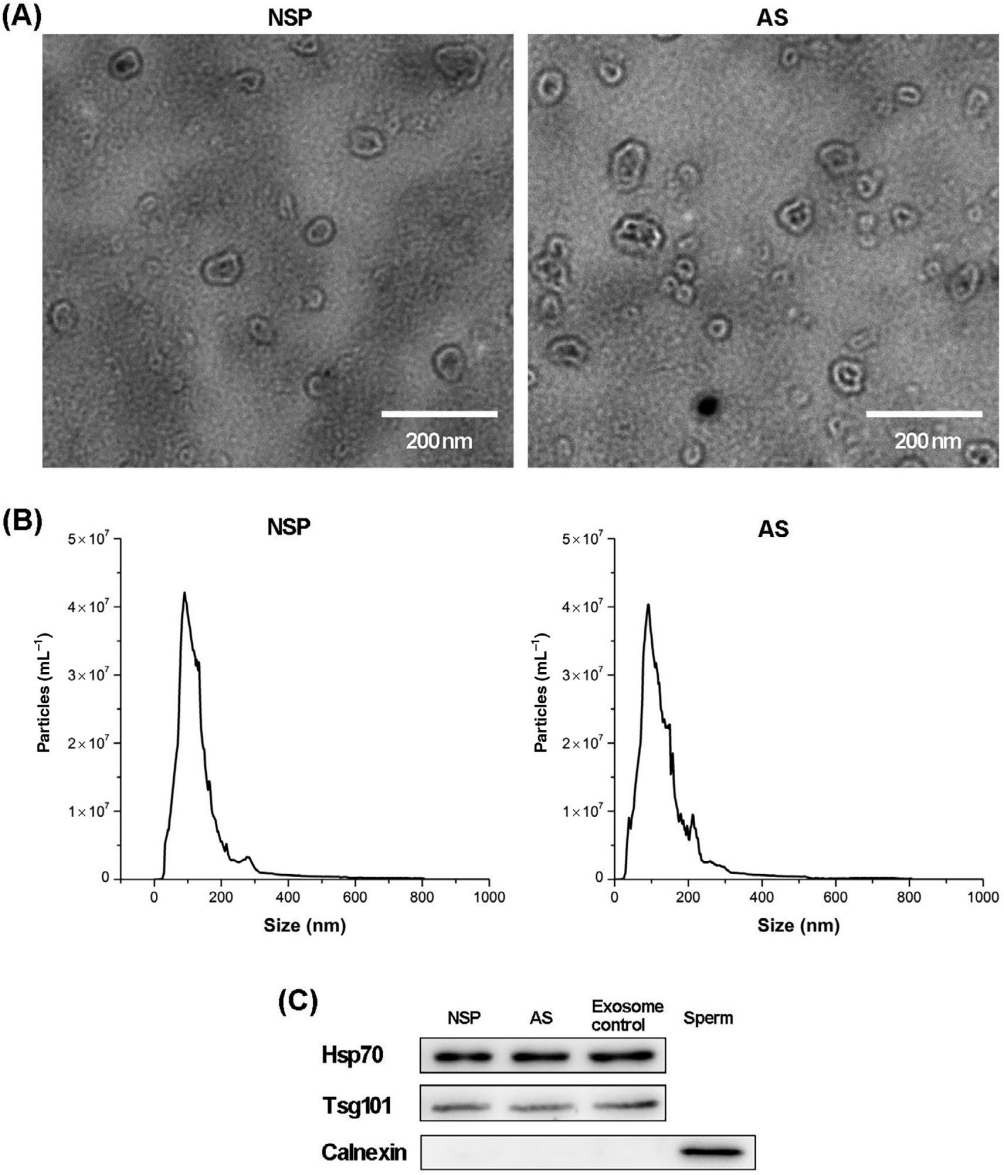
Characterization of seminal plasma exosomes. (A) Transmission electron microscopy images of exosomes isolated from normozoospermic (NSP) and asthenozoospermic (AS) men. Scale bars: 200 nm. (B) Representative nanoparticle tracking analysis plots of exosomes from NSP and AS men. (C) Western blot analysis of exosomal markers Hsp70 and Tsg101 and the cellular contamination marker Calnexin. Sperm cell lysate was included as a positive control for Calnexin.

Seminal plasma from asthenozoospermic patients seeking medical assistance for couples with infertility was collected to isolate and characterize semen exosomes. TEM analysis revealed the presence of round‐shaped vesicles with a typical cup‐shaped morphology and thin electron‐dense membranes, consistent with exosomal characteristics (Figure 1A). Nanoparticle tracking analysis (NTA) confirmed that the isolated vesicles primarily ranged in size from 50 to 150 nm, with a majority measuring less than 100 nm (Figure 1B). The vesicles from the two populations presented highly similar size distributions, with mean diameters of 97.56 nm for normozoospermic subjects and 98.22 nm for asthenozoospermic men. This size distribution aligns with the canonical range of exosomes (40–150 nm) and indicates minimal contamination from larger MVs (100–800 nm), supporting the effectiveness of sequential centrifugation protocol in enriching for sEVs. Vesicles derived from both normozoospermic and asthenozoospermic individuals displayed comparable morphologies, size distributions, and electron densities (Figure 1A,B). Western blot analysis further demonstrated the presence of the canonical exosomal markers Hsp70 and tumor susceptibility gene 101 protein (Tsg101) in the isolated vesicles (Figure 1C). Collectively, these data validate the successful isolation of exosomes from seminal plasma, with no apparent differences in exosome characteristics between the two study groups, suggesting that exosome production in seminal plasma is independent of sperm quality.

### 3.2 Analysis of miRNA Expression in Normozoospermic and Asthenozoospermic Seminal Plasma Exosomes

**FIGURE 2:**
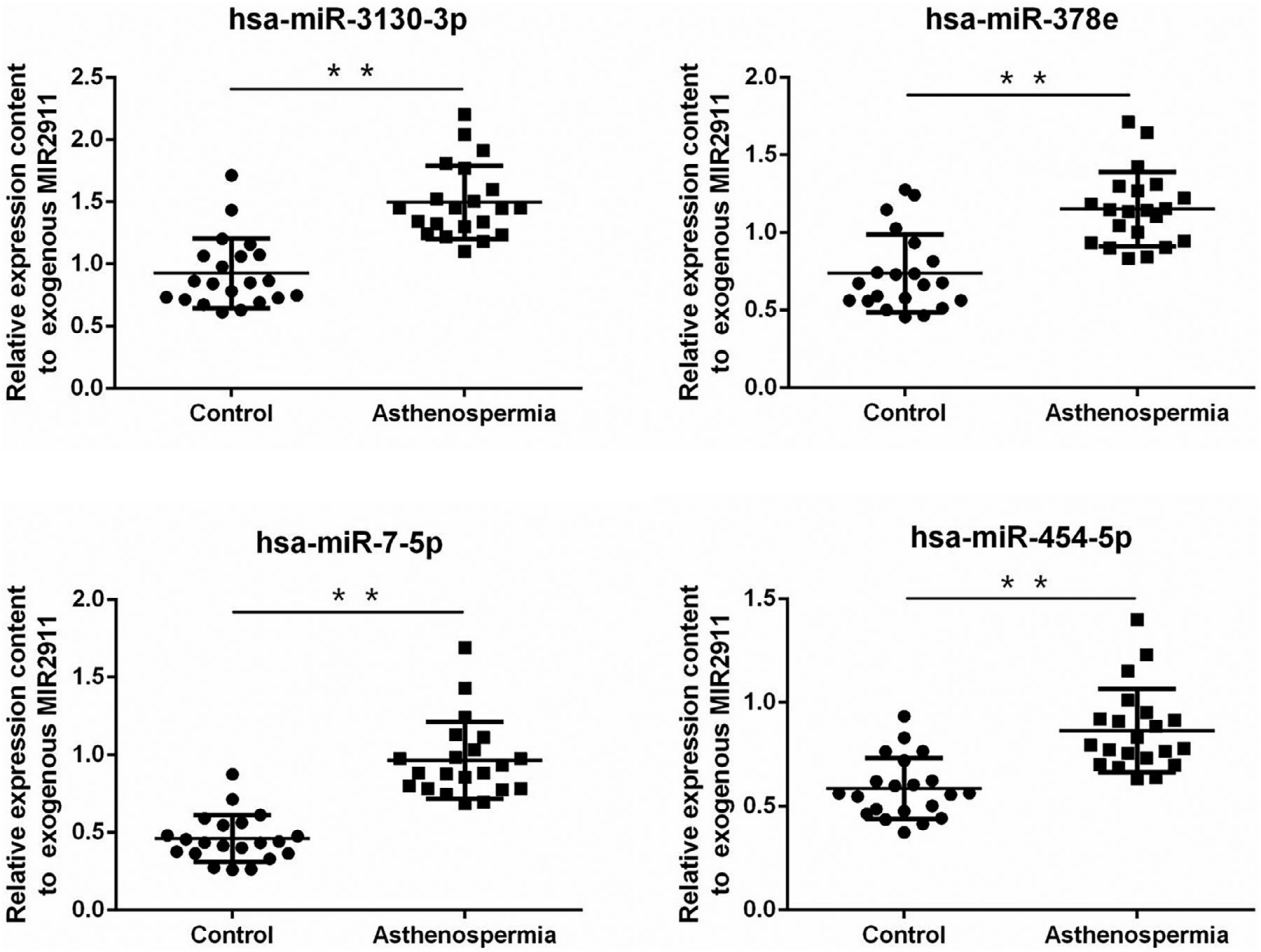
The relative levels of the four most significantly upregulated miRNAs (hsa‐miR‐3130‐3p, hsa‐miR‐378e, hsa‐miR‐7‐5p, and hsa‐miR‐454‐5p) in normal controls (*n* = 21) and asthenospermic patients (*n* = 21). Statistical significance was denoted by “*”for *p* < 0.05 and “**” for *p* < 0.01.

To identify candidate miRNAs in the seminal plasma exosomes of males with asthenozoospermia, we employed a pooled sequencing strategy. Exosome samples from all 21 individuals in the asthenozoospermia group were pooled, as were those from all 21 healthy controls. This approach was adopted to obtain a high‐concentration RNA input required for optimal library preparation and to identify miRNA expression patterns that are consistently altered at the group level, thereby facilitating initial biomarker discovery. The total RNA concentrations extracted from the case pool and control pool were 28.5 and 26.7 ng/µL, respectively. Analysis via UMI sequencing technology detected approximately 1000 miRNAs in both groups. Comparative analysis revealed 13 miRNAs exhibiting elevated expression in the exosomes of asthenozoospermic patients (Table 1). Analysis of isomiR expression patterns revealed that for the miRNA hsa‐miR‐7‐5p, which showed a notable increase in expression level, two isomiRs were detected in addition to its canonical sequence. Both sequence variants were expressed at relatively low levels and exhibited expression trends consistent with the canonical miRNA (File S2). To validate these screening results, we selected the four miRNAs with the most pronounced elevation (hsa‐miR‐3130‐3p, hsa‐miR‐378e, hsa‐miR‐7‐5p, and hsa‐miR‐454‐5p) for individual qRT‐PCR re‐screening and validation. This analysis unequivocally confirmed their significant elevation in seminal exosomes from asthenozoospermic individuals compared with normozoospermic controls (Figure 2). Although the fold‐change magnitudes differed from the sequencing data, likely due to distinct normalization methods and platform dynamics, the complete concordance in upregulation direction robustly affirms these miRNAs as bona fide differentially expressed markers.

**TABLE 1:** miRNAs with elevated expression in seminal plasma exosomes of patients with asthenospermia (based on pooled sequencing).

	Copy number		
Name	Control	Asthenozoospermia	Elevation/decrease
hsa‐miR‐3130‐3p	3	456	Elevation
hsa‐miR‐378e	32	1574	Elevation
hsa‐miR‐7‐5p	502	16,342	Elevation
hsa‐miR‐454‐5p	12	342	Elevation
hsa‐miR‐4433a‐5p	14	154	Elevation
hsa‐miR‐136‐3p	22	167	Elevation
hsa‐miR‐184	42	234	Elevation
hsa‐miR‐4423‐5p	33	112	Elevation
hsa‐miR‐143‐3p	184	504	Elevation
hsa‐miR‐6513‐5p	72	193	Elevation
hsa‐miR‐1287‐3p	54	118	Elevation
hsa‐miR‐185‐3p	76	133	Elevation
hsa‐miR‐6887‐3p	63	105	Elevation

### 3.3 Functional Annotation of the Predicted miRNA Targets

Using TargetScan software, we analyzed the 3′UTR sequences to predict potential target genes for the four significantly differentially expressed miRNAs (Files S3–S6). Considering that miRNAs must reach a certain expression level to effectively mediate target gene repression and thereby participate in the regulation of sperm motility, we prioritized the two highly expressed miRNAs, hsa‐miR‐7‐5p, and hsa‐miR‐378e, for subsequent functional characterization to investigate their potential roles in regulating sperm motility.

**FIGURE 3:**
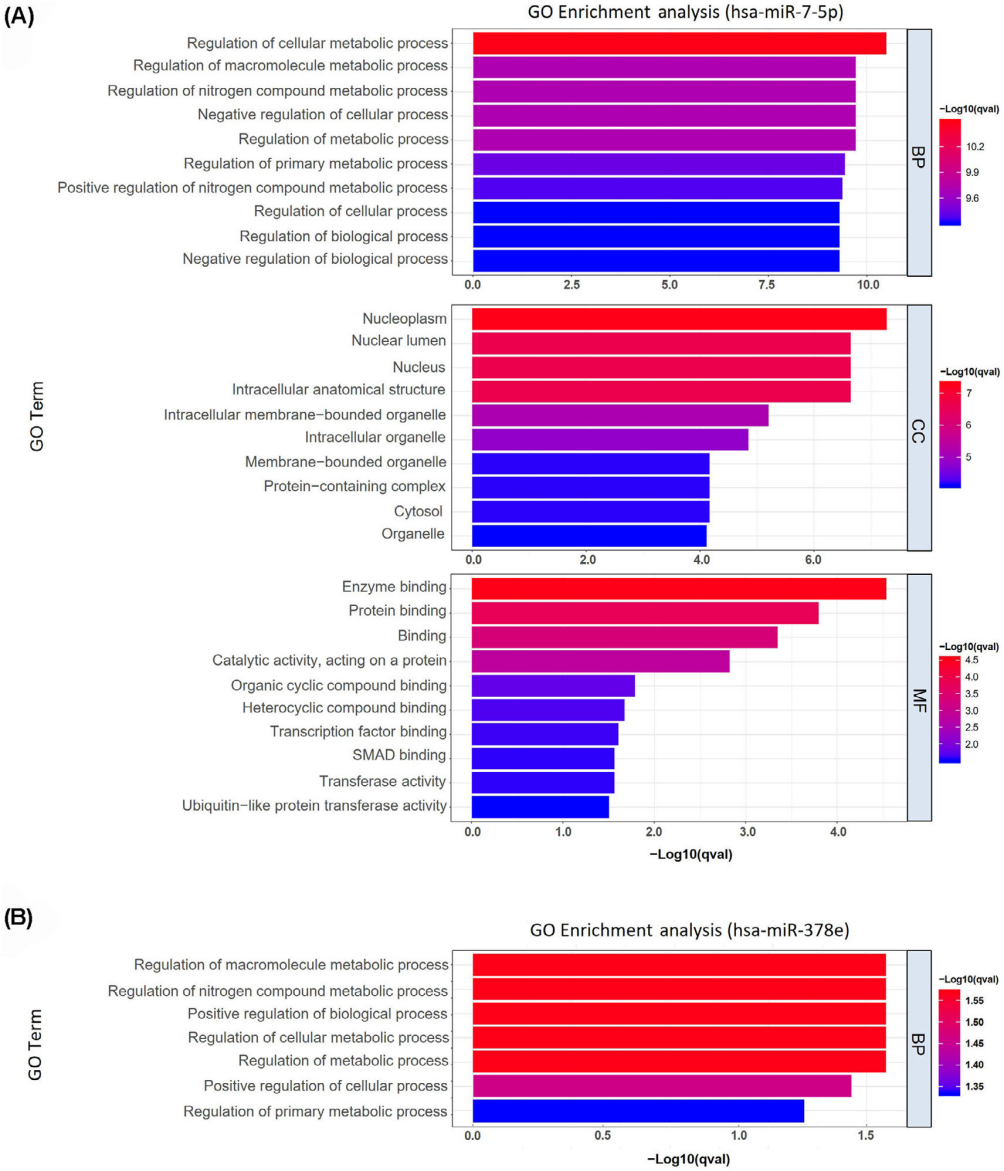
Functional enrichment analysis of GO terms for the predicted target genes of hsa‐miR‐7‐5p (A) and hsa‐miR‐378e (B): biological process (BP), cellular component (CC), and molecular function (MF) categories.

GO analysis was performed for the predicted target genes of both miRNAs, with the results detailed in Figure 3. The target genes of hsa‐miR‐7‐5p were significantly enriched across all three categories. In biological process (BP), the top terms were related to metabolic regulation (e.g., cellular, macromolecule, and nitrogen compound metabolic processes) and negative regulation of cellular processes. Cellular component (CC) analysis indicated a strong nuclear localization, with enrichment in the nucleoplasm, nuclear lumen, and nucleus. For molecular function (MF), enzyme binding was the most significant term. Conversely, for hsa‐miR‐378e, significant enrichment was confined to the BP category, where it shared several key regulatory terms with hsa‐miR‐7‐5p, including the regulation of macromolecule, nitrogen compound, and cellular metabolic processes, as well as positive regulation of BPs.

**FIGURE 4:**
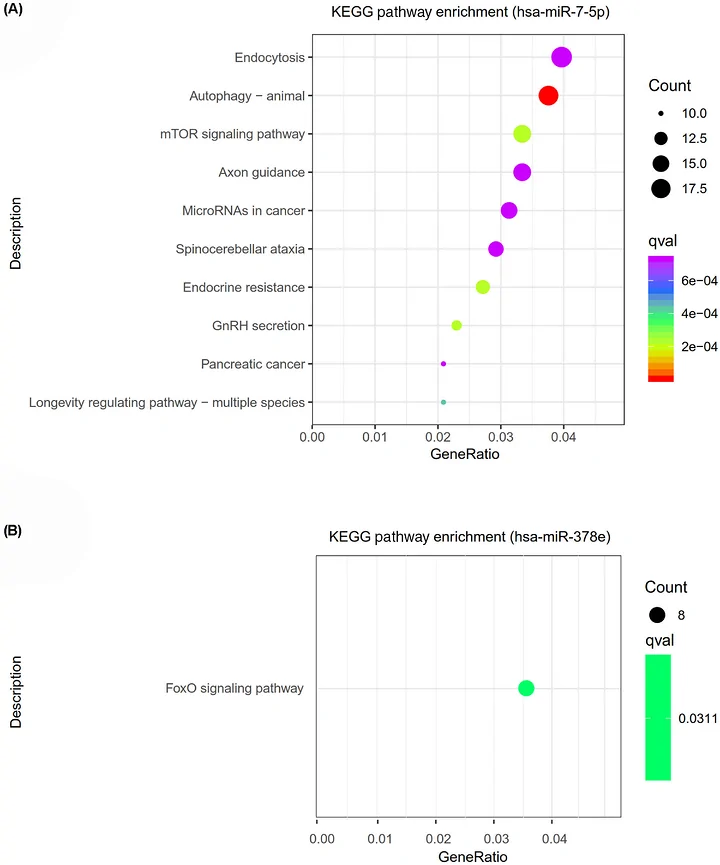
KEGG pathway enrichment analysis of potential target genes for hsa‐miR‐7‐5p (A) and hsa‐miR‐378e (B). The gene ratio represents the ratio of the number of genes enriched in this pathway to the total number of target genes.

Furthermore, KEGG pathway analysis was performed on the predicted target genes of hsa‐miR‐7‐5p and hsa‐miR‐378e to identify the biological pathways potentially regulated by these functional miRNAs. The results revealed that the candidate target genes of hsa‐miR‐7‐5p were significantly enriched in several key pathways related to cell differentiation, signal transduction, and hormone secretion, such as endocytosis, autophagy, the mTOR signaling pathway, axon guidance, miRNAs in cancer, endocrine resistance, and gonadotropin‐releasing hormone (GnRH) secretion (Figure 4A). These pathways are intricately connected to the regulatory mechanisms underlying spermatogenesis and sperm motility. In contrast, the candidate target genes of hsa‐miR‐378e were enriched only in the FoxO signaling pathway (Figure 4B).

### 3.4 Screening and Identification of hsa‐miR‐7‐5p Target Genes Related to the Regulation of Sperm Motility

**FIGURE 5:**
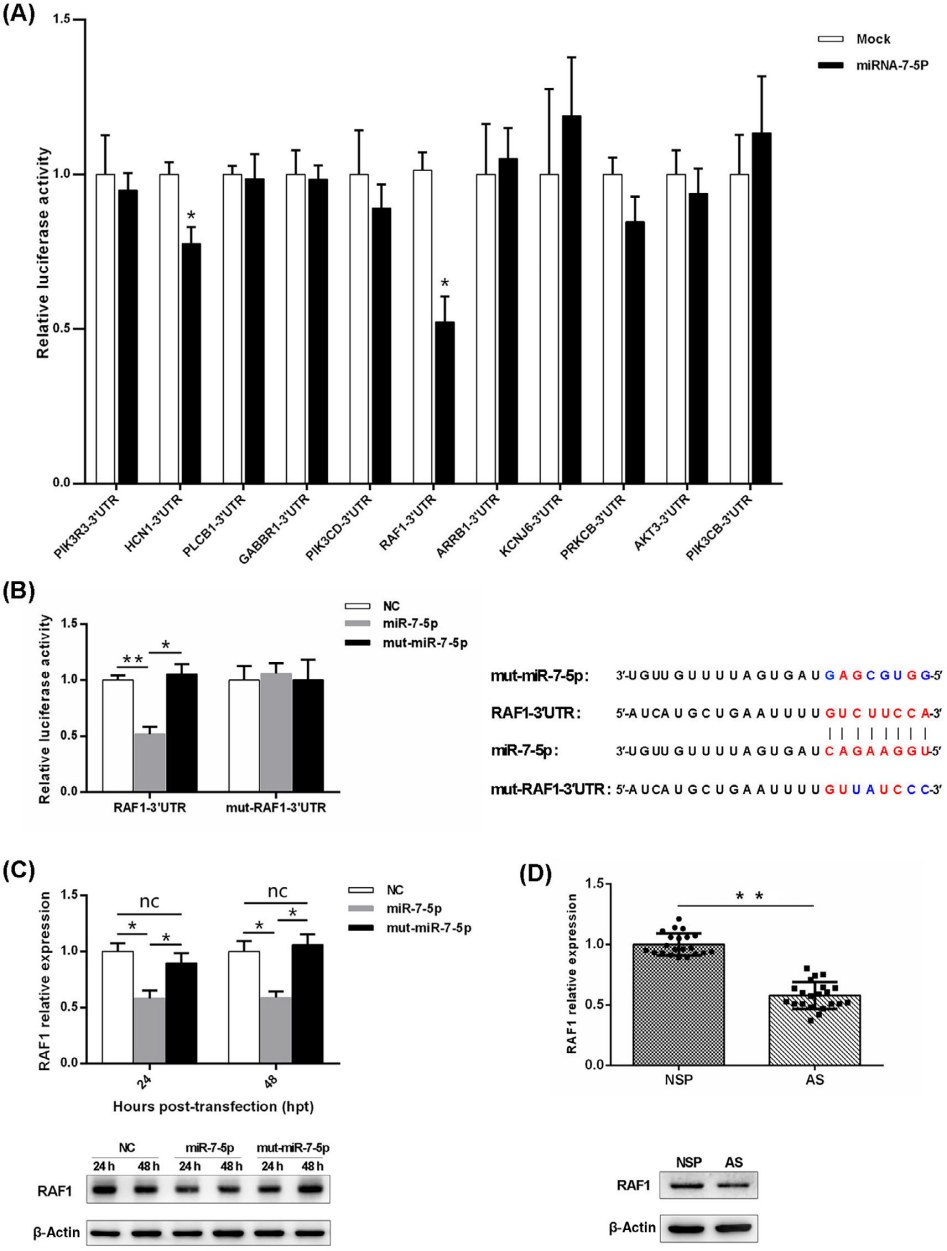
Identification of the involvement of hsa‐miR‐7‐5p in the regulation of candidate mRNA targets. (A) First round of DLRA for primary analysis of the interactions between hsa‐miR‐7‐5p and the 3'UTRs of eleven candidate genes enriched in the GnRH signaling pathway, including PIK3R3, HCN1, PLCB1, GABBR1, PIK3CD, RAF1, ARRB1, KCNJ6, PRKCB, AKT3, and PIK3CB. (B) DLRA was performed to confirm the interactions between hsa‐miR‐7‐5p and the 3′UTR of RAF1. The colored seed sequence of the miRNA or binding site of the 3′UTR and the corresponding mutants are shown on the right side. The wild‐type or mutated 3′UTR of RAF1 was cloned downstream of Renilla luciferase in the psiCHECK‐2 vector and then cotransfected into 293T cells with the miR‐7‐5p, mut‐miR‐7‐5p, or negative control (NC) mimics. Firefly and Renilla luciferase activities were measured at 48 h post‐transfection via a dual‐luciferase reporter system (Promega). Firefly luciferase served as the internal control. For each luciferase assay, the relative luciferase activity was normalized with respect to that of the NC. (C) mRNA and protein expression levels of RAF1 in EECs overexpressing hsa‐miR‐7‐5p. (D) Comparison of RAF1 mRNA and protein expression levels in sperm cells between the asthenozoospermic patient group and the healthy control group. Expression levels were normalized to β‐actin and are presented relative to the NSP group mean (set to 1). Statistical significance was denoted by “*”for *p* < 0.05 and “**” for *p* < 0.01.

To investigate the mechanism by which hsa‐miR‐7‐5p regulates sperm motility, eleven candidate genes enriched in the GnRH signaling pathway via KEGG analysis—PIK3R3, HCN1, PLCB1, GABBR1, PIK3CD, RAF1, ARRB1, KCNJ6, PRKCB, AKT3, and PIK3CB—were selected as priority targets for further investigation. Site‐directed dual luciferase reporter (DLRA) assays were conducted to evaluate the in vitro interactions between hsa‐miR‐M7‐5p and the 3′UTRs of these eleven candidates containing potential target sites. In the initial DLRA analysis, the relative luciferase activity of the RAF1 3′UTR reporter was significantly repressed by hsa‐miR‐7‐5p compared with that of the NC mimics (Figure 5A). To confirm that the downregulation of RAF1 by hsa‐miR‐7‐5p is sequence dependent, mutated hsa‐miR‐7‐5p mimics were utilized in a subsequent round of DLRA analysis (Figure 5B). These results indicated that mutating the seed sequence of hsa‐miR‐7‐5p abolished its repressive effect on the RAF1 reporter. To further validate the specificity of this repression, we constructed a corresponding 3′UTR reporter mutant, mut‐RAF1‐3′UTR. As anticipated, the DLRA analysis revealed that the mutated luciferase reporter no longer responded to hsa‐miR‐7‐5p (Figure 5B).

Given the central role of the epididymis in post‐testicular sperm maturation, we first assessed RAF1 expression in EECs overexpressing hsa‐miR‐M7‐5p. As shown in Figure 5C, qRT‐PCR analysis revealed that the overexpression of hsa‐miR‐M7‐5p significantly decreased RAF1 expression at both 24 and 48 hpt. Western blot analysis further confirmed the downregulation of RAF1 expression in EECs as a result of hsa‐miR‐M7‐5p overexpression. Subsequently, we further examined the expression levels of RAF1 in sperm cells from healthy control subjects (normozoospermic, sperm motility ≥32%) and asthenozoospermic patients (sperm motility <32%). qRT‐PCR and Western blot analysis revealed a significant decrease in RAF1 mRNA and protein expression in the asthenozoospermic group compared with the normozoospermic group (Figure 5D). These preliminary data suggest that hsa‐miR‐7‐5p is likely a functional miRNA that regulates sperm motility by targeting RAF1 expression.

### 3.5 Functional Exploration of hsa‐miR‐7‐5p Targeting RAF1

PPI analysis was performed using the STRING database for RAF1, and the resulting network was visualized in Cytoscape. The constructed network comprised 11 nodes and 50 edges, and functional annotation clustering indicated that the RAF1‐centered PPI network is involved in several key BPs, including the MAPK cascade, GnRH secretion, Ras protein signal transduction, and the PI3K–Akt signaling pathway (Figure S1).

**FIGURE 6:**
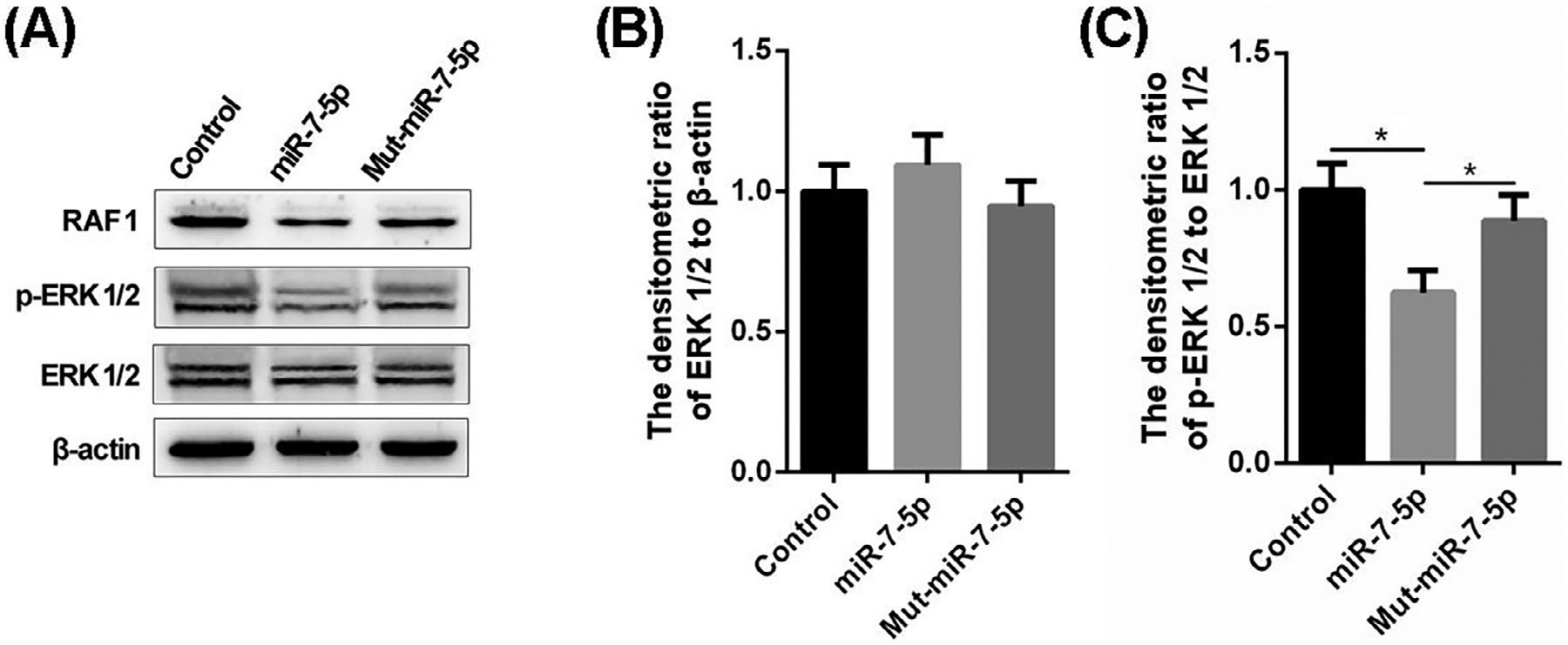
Effect of hsa‐miR‐7‐5p‐mediated RAF1 inhibition on total ERK1/2 and phosphorylated ERK1/2 (p‐ERK1/2) expression in sperm cells. (A) Representative Western blot analysis showing the effects of hsa‐miR‐7‐5p‐targeted RAF1 suppression on p‐ERK1/2 and total ERK1/2 protein levels. (B) The densitometric ratio of ERK1/2 normalized to β‐actin. No significant differences were observed between the different groups. Data are presented as mean ± SEM from three independent experiments (*n* = 3). (C) The densitometric ratio of p‐ERK1/2 to total ERK1/2 ratio. Sperm cells treated with hsa‐miR‐7‐5p showed significantly reduced p‐ERK1/2 expression compared with controls (*p < 0.05). Data represent mean ± SEM from three independent experiments (*n* = 3).

The RAF1 kinase is a well‐documented regulator of male reproduction, with established expression in the testis and a functional role in epididymal sperm maturation [28, 29]. As a central component of the MAPK pathway, which is critical for sperm motility and capacitation [30], RAF1 represents a plausible mechanistic link through which exosomal miRNAs may influence sperm function. To further elucidate this pathway, we examined the effect of miR‐7‐5p‐mediated RAF1 suppression on its downstream effector ERK1/2 in the MAPK signaling cascade. As shown in Figure 6, while total ERK1/2 protein levels remained unaffected, miR‐7‐5p‐induced suppression of RAF1 significantly inhibited ERK1/2 phosphorylation in sperm cells.

## 4 Discussion

Seminal plasma exosomes act as crucial mediators of sperm development and can increase sperm motility and induce capacitation [13, 31]. These nanovesicles transport functional proteins and miRNAs that actively facilitate sperm maturation, equipping spermatozoa with progressive motility and the capacity to fuse with oocytes [32, 33, 34]. Accumulating evidence indicates that aberrant sperm motility is associated with dysregulated miRNA expression patterns during spermatogenesis, potentially leading to impaired sperm development [35, 36]. In this study, we utilized UMI‐based absolute quantification sequencing to systematically characterize miRNA profiles in seminal plasma exosomes derived from both healthy controls and patients with asthenozoospermia. Our analysis identified approximately 1000 miRNAs, and differential expression analysis revealed 13 miRNAs with elevated expression in asthenozoospermic individuals relative to normozoospermic controls. These candidate miRNAs in seminal plasma exosomes represent potential biomarkers that go beyond routine diagnosis, offering mechanistic insights into idiopathic asthenozoospermia and highlighting novel therapeutic targets.

Exosomes are sEVs that are typically 30–150 nm in diameter, are secreted by virtually all cell types, and are found in a wide range of biological fluids. In the present study, exosomes isolated from seminal plasma via ultracentrifugation exhibited a characteristic bilayered saucer‐like morphology. NTA revealed that exosomes derived from the seminal plasma of both asthenozoospermic patients and healthy controls presented similar morphological features, with average particle sizes ranging from 90 to 100 nm. Additionally, Western blot analysis confirmed the presence of canonical exosomal marker proteins, including Hsp70 and Tsg101, in the isolated vesicles. Collectively, these results confirm the successful isolation of exosomes from seminal plasma and highlight their abundant presence in semen.

Multiple studies on seminal plasma exosomal miRNAs have elucidated their crucial roles in male infertility. MiR‐34b‐5p has been identified as a biomarker for reduced male fertility, with its expression levels in normal seminal plasma exosomes being significantly higher than those observed in patients with asthenozoospermia or oligozoospermia [37]. Furthermore, seminal plasma exosome‐derived miR‐26‐5p has been shown to promote M2 polarization of decidual macrophages by targeting the PTEN/PI3K/AKT signaling pathway, thereby establishing an immune‐tolerant microenvironment conducive to embryo implantation and development [38]. Studies have demonstrated that miR‐765 and miR‐1275 are markedly upregulated in the seminal plasma exosomes of patients with spermatorrhea, showing distinct associations with male infertility and varicocoele, respectively [39, 40]. Zhu et al. reported that miR‐184 expression was significantly elevated in asthenozoospermic patients compared with normozoospermic controls, with its levels positively correlated with sperm concentration but inversely correlated with the percentage of progressively motile spermatozoa [41]. Additionally, Ma et al. demonstrated that miR‐210‐3p was highly expressed in the exosomes of varicocoele patients, with its levels significantly decreasing following microsurgical varicocoelectomy [42]. In addition to human studies, miRNAs have been consistently detected in seminal plasma exosomes of various mammalian species, including bulls and boars [43, 44]. These miRNAs play pivotal roles in intercellular communication between parental cells and their microenvironment through RNA‐mediated mechanisms, ultimately influencing critical sperm functions such as motility.

Our comparative analysis of seminal plasma exosomal miRNA profiles between healthy controls and asthenozoospermic patients revealed a consistent upregulation pattern, with all 13 differentially expressed miRNAs showing increased expression levels in the patient group. This global elevation in miRNA expression in asthenozoospermic patients aligns with previous findings in oligozoospermic patients, where the upregulation of seminal plasma miRNAs predominates over their downregulation. Through qRT‐PCR validation, we identified four miRNAs (hsa‐miR‐3130‐3p, hsa‐miR‐378e, hsa‐miR‐7‐5p, and hsa‐miR‐454‐5p) that were significantly upregulated in asthenozoospermic seminal plasma exosomes. Notably, hsa‐miR‐7‐5p presented remarkably high copy numbers in patient samples, strongly suggesting its potential as a key regulator of sperm motility.

Further bioinformatics analysis revealed 558 potential target genes for hsa‐miR‐7‐5p. Functional annotation demonstrated that these targets were significantly enriched in fundamental BPs, including cellular metabolism, macromolecule metabolism, and nitrogen compound metabolism. Pathway analysis further revealed their predominant involvement in several critical signaling pathways, including endocytosis, autophagy, mTOR signaling, axon guidance, endocrine resistance, and GnRH secretion. Notably, the PI3K/AKT/mTOR signaling pathway was identified as a key regulator throughout all stages of spermatogenesis [45, 46]. This is supported by the findings of Boyer et al., who reported that conditional knockout of mTOR in Sertoli cells led to disintegration of the spermatogenic epithelium, alterations in Sertoli cell polarity, increased germ cell apoptosis, shedding of immature germ cells, a reduced sperm count in the epididymis, and an increased prevalence of abnormal spermatozoa in adult mice, ultimately resulting in infertility [47]. Consistent evidence from human, rat, and bovine studies has confirmed the beneficial effects of insulin and insulin‐like growth factors on sperm motility [48, 49, 50], with PI3K/AKT/mTOR serving as the canonical pathway mediating insulin signaling. Mechanistically, insulin likely activates the PI3K/AKT/mTOR cascade through IRS1 phosphorylation, thereby modulating sperm motility parameters. GnRH, synthesized and secreted in a pulsatile manner by the hypothalamus, functions as a master regulator of reproductive function. Its pulsatile secretion pattern stimulates the synthesis and release of gonadotropins, luteinizing hormone and follicle‐stimulating hormone, which subsequently act on target organs to promote testosterone, progesterone and estrogen production. This endocrine cascade is essential for both spermatogenesis and the enhancement of sperm motility. Notably, GO and KEGG enrichment analyses of the predicted targets for another significantly differentially expressed miRNA, hsa‐miR‐378e, revealed a striking convergence with those of hsa‐miR‐7‐5p across multiple pivotal BPs. This overlap suggests that these two miRNAs may function cooperatively by fine‐tuning a core regulatory network, ultimately contributing to the pathogenesis of asthenozoospermia. Future studies are warranted to dissect their synergistic roles and the precise molecular mechanisms underlying their coordination in spermatogenesis.

To further elucidate the molecular mechanisms by which seminal plasma exosomal miRNAs regulate sperm motility, this study confirmed through dual‐luciferase reporter assays that hsa‐miR‐7‐5p specifically targets RAF1, a key component of the GnRH signaling pathway. PPI analysis indicated that RAF1 is predominantly involved in regulating GnRH secretion, MAPK signaling, Ras protein signal transduction and the PI3K‒Akt signaling pathway. At the molecular level, RAF1 serves as a MAP kinase kinase kinase that, upon activation by Ras family GTPases, initiates the MAPK cascade to control essential BPs such as cell proliferation, differentiation, and survival. Notably, among the pathways associated with sperm motility, the MAPK signaling pathway is one of the most frequently reported and affects sperm function through the modulation of mature sperm flagellar movement, hyperactivation, and the acrosome reaction [51]. Previous studies have demonstrated that molecules such as arachidonic acid and HSP90 regulate sperm motility, the acrosome reaction, and capacitation through the P38 MAPK signaling pathway [52, 53]. Our findings suggest that hsa‐miR‐7‐5p may regulate the MAPK signaling pathway by directly targeting RAF1, thereby affecting sperm motility. Notably, the differential expression of hsa‐miR‐7‐5p isomiRs detected in seminal plasma exosomes from asthenospermic patients warrants special emphasis. This finding is consistent with emerging evidence demonstrating that both miRNAs and their isomiR variants exhibit dysregulation in the semen of infertile males [24]. Previous studies have suggested that certain isomiRs may differ from their canonical counterparts in aspects such as target selection, miRNA stability, and incorporation into the RNA‐induced silencing complex, thus coordinately regulating relevant pathways [54, 55]. Consequently, future research should delve deeper into the influence of isomiRs in seminal plasma exosomes on the target regulatory function of canonical hsa‐miR‐7‐5p, as well as their potential biological roles in modulating sperm motility.

Our findings suggest that hsa‐miR‐7‐5p may regulate the MAPK signaling pathway by directly targeting RAF1, thereby affecting sperm motility. Nevertheless, it is important to acknowledge the limitations of this study. The validation of differentially expressed miRNAs via qRT‐PCR was conducted on the same sample set used for the initial RNA‐seq discovery phase. While this provides robust technical validation of our sequencing data, it does not constitute a validation of the biomarker's clinical utility on an independent cohort. Therefore, our results for hsa‐miR‐7‐5p should be interpreted as promising preliminary findings. Future studies involving larger, independent patient cohorts are essential to definitively confirm the usefulness of hsa‐miR‐7‐5p as a non‐invasive biomarker for asthenozoospermia.

## 5 Conclusions

Our study systematically characterized the miRNA expression profiles of seminal plasma exosomes from healthy controls and asthenozoospermic patients and identified 13 differentially expressed miRNAs. Bioinformatics analysis indicated that hsa‐miR‐7‐5p, which is highly expressed in the seminal plasma of asthenozoospermic individuals, may impair sperm function by modulating critical pathways such as mTOR signaling, endocrine resistance, and GnRH secretion. Notably, dual‐luciferase reporter assays confirmed that hsa‐miR‐7‐5p directly targets RAF1, suggesting its potential role in regulating sperm motility through RAF1‐mediated MAPK signaling networks. Nevertheless, these findings warrant further validation through functional experiments.

## Author Contributions

Zhi‐Jian Zhu and En‐Zhong Li contributed to the design of this work. Zhi‐Jian Zhu, En‐Zhong Li, and Jin‐Feng Li contributed to the data analysis and interpretation. Zhi‐Jian Zhu, Meng‐Li Cui, Wen‐Jing Yuan, Xi‐Qiao Yao, Ruo‐Qing Li, Jia‐Ning Zhang, and Shuo Zhang contributed to the result validation. Zhi‐Jian Zhu, En‐Zhong Li, Meng‐Li Cui, Xi‐Qiao Yao, Ruo‐Qing Li, and Jin‐Feng Li contributed to the drafting and editing of this article. All the authors have read and agreed to the published version of the manuscript.

## Conflicts of Interest

The authors declare no conflicts of interest.

## Supporting information




**Supplementary File 1**: The sperm parameters for both the asthenozoospermic patient group (AS) and the normal control group (NC).

**Supplementary File 2**: Hsa‐miR‐7‐5p isomiR expression patterns from UMI‐based transcriptome sequencing.

**Supplementary File 3**: Predicted target genes of hsa‐miR‐7‐5p from TargetScan analysis.

**Supplementary File 4**: Predicted target genes of hsa‐miR‐378e from TargetScan analysis.

**Supplementary File 5**: Predicted target genes of hsa‐miR‐454‐5p from TargetScan analysis.

**Supplementary File 6**: Predicted target genes of hsa‐miR‐3130‐3p from TargetScan analysis.

**Supplementary Figure 1**: Protein‒protein interaction (PPI) network analysis of RAF1. The PPI network centered on RAF1 is associated with GnRH secretion, the MAPK cascade, Ras protein signal transduction and the PI3K–Akt signaling pathway. Nodes are color‐coded: red (RAF1), blue (proteins related to the GnRH signaling pathway), and gray (other RAF1‐interacting proteins). Direct interactions are indicated by gray connecting lines.

**Supplementary File 1**: andr70159‐sup‐0008‐SuppMat.docx

## Data Availability

The data that support the findings of this study are available from the corresponding author upon reasonable request.
