# Supplementary material for: Seminal Plasma Exosomal miRNA Profiling Reveals hsa‐miR‐7‐5p as a Key Regulator of Sperm Motility in Asthenozoospermia
Source: Andrology. 2025 Dec 24;14(7):2166–79. doi: 10.1111/andr.70159 (PMC13592056; doi:10.1111/andr.70159)
Supplement: Supplementary file 1 [file ANDR-14-2166-s004.docx]

Supplementary figures:

Supplementary Figure 1. Protein‒protein interaction (PPI) network analysis of RAF1. The PPI network centered on RAF1 is associated with GnRH secretion, the MAPK cascade, Ras protein signal transduction and the PI3K-Akt signalling pathway. Nodes are color-coded: red (RAF1), blue (proteins related to the GnRH signalling pathway), and gray (other RAF1-interacting proteins). Direct interactions are indicated by gray connecting lines.
