# Supplementary material for: Seminal Plasma Exosomal miRNA Profiling Reveals hsa‐miR‐7‐5p as a Key Regulator of Sperm Motility in Asthenozoospermia
Source: Andrology. 2025 Dec 24;14(7):2166–79. doi: 10.1111/andr.70159 (PMC13592056; doi:10.1111/andr.70159)
Supplement: Supplementary file 2 [file ANDR-14-2166-s005.docx]

Supplementary table 1: Hsa-miR-7-5p isomiR expression patterns from UMI-based transcriptome sequencing.

| miRNA | Variant | Sequence | Seq length | Control | Asthenozoospermia |
| --- | --- | --- | --- | --- | --- |
| hsa-miR-7-5p | NA | TGGAAGACTAGTGATTTTGTTGTT | 23 | 485 | 15418 |
| hsa-miR-7-5p | iso_3p:-1 | TGGAAGACTAGTGATTTTGTTGT | 22 | 23 | 923 |
| hsa-miR-7-5p | iso_3p:-2 | TGGAAGACTAGTGATTTTGTTG | 21 | 4 | 166 |
