# Supplementary material for: Seminal Plasma Exosomal miRNA Profiling Reveals hsa‐miR‐7‐5p as a Key Regulator of Sperm Motility in Asthenozoospermia
Source: Andrology. 2025 Dec 24;14(7):2166–79. doi: 10.1111/andr.70159 (PMC13592056; doi:10.1111/andr.70159)
Supplement: Supplementary file 3 [file ANDR-14-2166-s008.docx]

Supplementary file legends

Supplementary file 1: The sperm parameters for both the asthenozoospermic patient group (AS) and the normal control group (NC).

Supplementary file 2: Hsa-miR-7-5p isomiR expression patterns from UMI-based transcriptome sequencing.

Supplementary file 3: Predicted target genes of hsa-miR-7-5p from TargetScan analysis.

Supplementary file 4: Predicted target genes of hsa-miR-378e from TargetScan analysis.

Supplementary file 5: Predicted target genes of hsa-miR-454-5p from TargetScan analysis.

Supplementary file 6: Predicted target genes of hsa-miR-3130-3p from TargetScan analysis.
